# Hepatic oxidative DNA damage is associated with increased risk for hepatocellular carcinoma in chronic hepatitis C

**DOI:** 10.1038/sj.bjc.6604204

**Published:** 2008-01-29

**Authors:** H Tanaka, N Fujita, R Sugimoto, N Urawa, S Horiike, Y Kobayashi, M Iwasa, N Ma, S Kawanishi, S Watanabe, M Kaito, Y Takei

**Affiliations:** 1 Department of Gastroenterology and Hepatology, Division of Clinical Medicine and Biomedical Science, Institute of Medical Sciences, Mie University Graduate School of Medicine, Mie, Japan; 2 Department of Anatomy, Mie University Graduate School of Medicine, Mie, Japan; 3 Faculty of Health Science, Suzuka University of Medical Science, Mie, Japan; 4 Center for Physical and Mental Health, Mie University, Mie, Japan

**Keywords:** oxidative stress, free radicals, 8-hydroxydeoxyguanosine, iron, hepatitis C virus, immunohistochemistry

## Abstract

Although the oxidative stress frequently occurs in patients with chronic hepatitis C, its role in future hepatocellular carcinoma (HCC) development is unknown. Hepatic 8-hydroxydeoxyguanosine (8-OHdG) was quantified using liver biopsy samples from 118 naïve patients who underwent liver biopsy from 1995 to 2001. The predictability of 8-OHdG for future HCC development and its relations to epidemiologic, biochemical and histological baseline characteristics were evaluated. During the follow-up period (mean was 6.7±3.3 years), HCC was identified in 36 patients (30.5%). Univariate analysis revealed that 16 variables, including 8-OHdG counts (65.2±20.2 *vs* 40.0±23.5 cells per 10^5^ *μ*m^2^, *P*<0.0001), were significantly different between patients with and without HCC. Cox proportional hazard analysis showed that the hepatic 8-OHdG (*P*=0.0058) and fibrosis (*P*=0.0181) were independent predicting factors of HCC. Remarkably, 8-OHdG levels were positively correlated with body and hepatic iron storage markers (*vs* ferritin, *P*<0.0001 *vs* hepatic iron score, *P*<0.0001). This study showed that oxidative DNA damage is associated with increased risk for HCC and hepatic 8-OHdG levels are useful as markers to identify the extreme high-risk subgroup. The strong correlation between hepatic DNA damage and iron overload suggests that the iron content may be a strong mediator of oxidative stress and iron reduction may reduce HCC incidence in patients with chronic hepatitis C.

Hepatocellular carcinoma (HCC) is one of the most common malignancies worldwide, and the death rate due to this tumour has been increasing over the past 20–30 years in the Unites States (El-Serag, 2002) and in Japan (Nishioka *et al*, 1991). Chronic infection with hepatitis C virus (HCV) has been recognised as an increased risk of HCC; approximately 20% of HCV-infected individuals have diseases that progress to cirrhosis, and about 40% of these patients develop HCC after a mean of 10–15 years (Seeff, 2002). Consequently, surveillance programmes based on periodic ultrasound examination and serum *α*-fetoprotein determination are recommended for patients with chronic hepatitis C. However, the effectiveness of these protocols has not been fully assessed and they afford no contribution to improvement of clinical outcomes in patients with chronic hepatitis C (Gebo *et al*, 2002). Therefore, it is desirable to establish a useful marker that could identify cases at high risk of developing HCC among chronic HCV-infected patients.

Although the mechanisms underlying HCC development during chronic HCV infection have been widely investigated, they are still unclear. It has been reported that structural and nonstructural proteins of HCV, such as Core and NS3, play a role in cell transformation, using *in vitro* cell culture systems (Sakamuro *et al*, 1995; Ray *et al*, 1996) and transgenic animals (Moriya *et al*, 1998). But whether expression of HCV protein(s) directly induces HCC in chronic HCV-infected patients is unknown. Recently, it has been assumed that oxidative stress may be relevant to this process of HCV-induced carcinogenesis, as has been suggested in several other malignancies (Kasai, 1997). Considerable data suggest that reactive oxygen species (ROS) may play a pathogenic role in carcinogenesis (Crawford and Cerutti, 1985). The most damaging species among the many ROS is the hydroxyl radical. The hydroxyl radical has been shown to be responsible for a number of base modifications that include thymine glycol, thymidine glycol (Cathcart *et al*, 1984), 5-(hydroxylmethyl)uracil (Hollstein *et al*, 1984), and also 8-hydroxydeoxyguanosine (8-OHdG) (Shigenaga *et al*, 1989; Kasai, 1997). 8-Hydroxydeoxyguanosine is a modification of guanine that induces a point mutation in the daughter DNA strands (Kuchino *et al*, 1987; Shibutani *et al*, 1991) and it is therefore used as a marker of oxidatively generated DNA damage in several diseases (Shigenaga *et al*, 1989; Kasai, 1997). In patients with chronic hepatitis C, increased 8-OHdG in DNA extracted from liver tissue was also reported (Shimoda *et al*, 1994; Mahmood *et al*, 2004; Fujita *et al*, 2007). These reports suggest that oxidative stress may be involved in the progression of liver disease, but they showed no direct participation of oxidative stress in hepatocarcinogenesis in the liver of HCV-infected patients. Also, no information is available on whether ROS-mediated damage to DNA is useful for prediction of HCC development in chronic hepatitis C patients.

In view of these considerations, we have examined the influence of the degree of ROS-mediated hepatic DNA damage as measured by counts of 8-OHdG immunohistochemically positive hepatocyte nuclei on the prevalence of future HCC development in chronic HCV-infected patients. Moreover, we evaluated the relation of the degree of ROS-mediated DNA damage with the epidemiologic, biochemical, and histological findings in chronic hepatitis C.

## PATIENTS AND METHODS

### Patients with chronic hepatitis C

This study comprised 118 consecutive patients (66 males and 52 females; mean age 55.8±10.8 years) recruited between January 1995 and October 2001 with HCV-related chronic hepatitis (Table 1). All patients fulfilled the following inclusion criteria: (1) liver injury caused by chronic HCV infection. All patients had persistently elevated serum alanine aminotransferase (ALT) levels and were seropositive for both anti-HCV antibody (the third-generation enzyme-linked immunosorbent assay; Ortho Diagnostic Systems, Raritan, NJ, USA) and HCV-RNA (Amplicor-HCV assay; Roche Molecular Diag. Co., Tokyo, Japan). (2) Liver biopsy. Liver tissue was obtained by percutaneous needle biopsy in all patients for diagnostic purposes. (3) Follow-up without interferon (IFN)-based therapy. The follow-up consisted of monthly blood tests and monitoring of tumour markers at the outpatients clinic of our department, and ultrasonography and dynamic computed tomography were performed at regular intervals. As it is known that IFN treatment reduces the incidence of HCC in patients with chronic hepatitis C (Nishiguchi *et al*, 1995), patients with a history of previous IFN-based treatment were excluded from the study. None of them had received any antiviral therapy during the follow-up period.

Exclusion criteria were as follows: a family history of haemochromatosis; haemolytic disease; serological markers for hepatitis B virus (HBV) (hepatitis B surface antigen and hepatitis B core antibody); or human immunodeficiency virus infection. Patients with concurrent diseases or those taking medications that may interfere with free radical production, such as nonsteroidal anti-inflammatory drugs, vitamins and iron-containing drugs, were excluded from the study. Patients with chronic alcohol consumption of ethanol in excess of 40 g day^−1^ for male and 20 g day^−1^ for female for at least 5 years were also excluded. All patients had no HCC or other cancers, by an initial screening examination. Informed consent was obtained from each patient and the study was approved by the Ethical Committee of Mie University. The study was carried out according to the ethical guidelines of the 1975 Declaration of Helsinki.

Clinical parameters were obtained for each patient at the time of liver biopsy: age; sex; body mass index; duration of HCV infection (when contamination was very probable and a precise data; transfusion or drug addiction in the past year); alcohol intake; biochemical, haematological, iron-related, and virological serum markers; and liver histological findings and 8-OHdG immunoreactivity.

The diagnosis of HCC was made by several imaging methods (ultrasonography, dynamic computed tomography, arteriography, or magnetic resonance imaging) and confirmed histologically in 22 cases. Time to HCC occurrence was defined as the interval between the date of liver biopsy and the detection of tumour, death without HCC occurrence, or the last examination until 31 October 2006. All patient deaths were considered end points irrespective of cause of death. The mean follow-up period was 6.7±3.3 (range, 0.4–11.8) years.

### Histological evaluation

All of the liver biopsy samples were stained with haematoxylin–eosin and Masson's trichrome, and were graded for the degree of necroinflammatory activity and staged for the extent of fibrosis using the criteria of Desmet *et al* (1994). The histological quantification of hepatic iron was carried out according to Deugnier *et al* (1992) by scoring iron separately within hepatocytes (hepatic iron score, 0–36), sinusoidal cells (sinusoidal iron score, 0–12), and portal tracts or fibrotic tissue (portal iron score, 0–12) using liver samples stained with Perls’ Prussian blue. The total iron score (TIS, 0–60) was defined by the sum of these scores. This score has been shown to highly correlate with the biochemical hepatic iron index and hepatic iron concentration as measured by the atomic absorption spectrophotometry in patients with chronic liver diseases (Piperno *et al*, 1998; Silvia *et al*, 2005).

### Immunohistochemical study

Immunohistochemical staining of 8-OHdG was performed as previously described (Fujita *et al*, 2007). Mouse monoclonal antibody against 8-OHdG (Japanese Aging Control Institute, Shizuoka, Japan) and Alexa 488-labelled goat antibody against mouse IgG (Molecular Probes, Eugene, OR, USA) were used. The degree of immunoreactivity was estimated by counting the number of stained hepatocyte nuclei using Adobe Photoshop version 5.5 and NIH Image free software (Vers. 1.62, National Institute of Health, image program).

### Statistical analysis

Results were expressed as mean±s.d. or median. Comparisons between groups were performed using the Mann–Whitney *U*-test or Kruskal–Wallis test for continuous variables and the *χ*^2^ or Fisher’s exact test for categorical data. Correlation coefficients between numerical variables were calculated as Spearman’s rank test. Cumulative HCC incidence curves were determined using the Kaplan–Meier method and the differences between groups were assessed with the log-rank test. Cox proportional hazard regression analysis was used to identify significant factors that influence future HCC development. All tests were two-tailed, and *P*-values less than 0.05 were considered as statistically significant. Statistical analyses were performed using the SPSS 11.5 software (SPSS Inc., Chicago, IL, USA).

## RESULTS

### *In situ* detection of 8-OHdG-positive hepatocytes using biopsy samples

**Figure 1:**
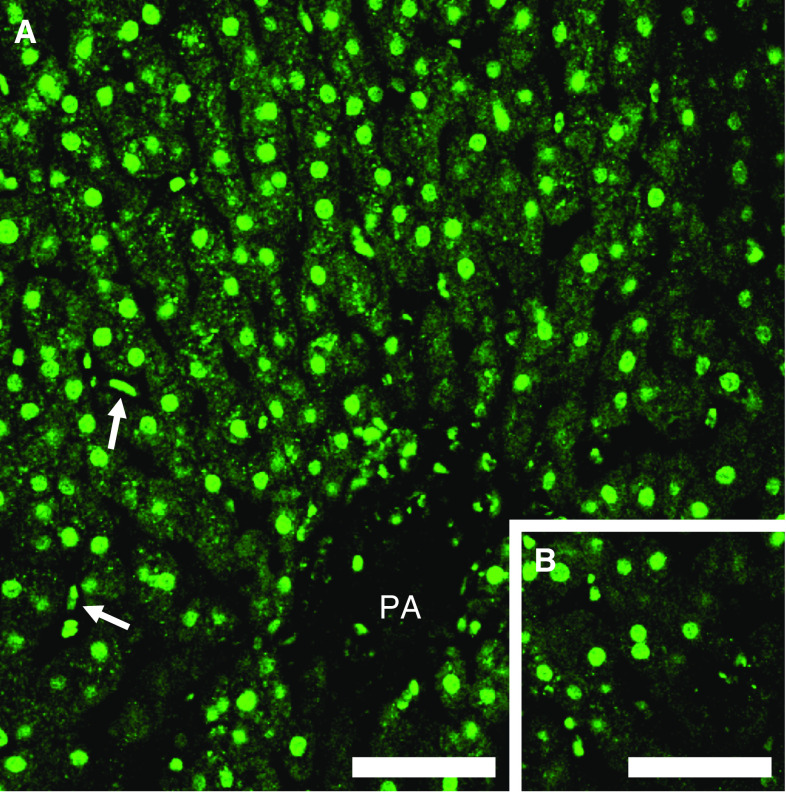
8-Hydroxydeoxyguanosine immunohistochemical staining in liver tissue from chronic hepatitis C and control (simple fatty liver) patients. (**A**) In the liver of chronic hepatitis C patient, 8-OHdG immunoreactivity was strongly observed throughout the whole acinus (PA=portal area) and mainly in the nuclei of hepatocytes and Kupffer cells (arrows in (**A**)). (**B**) In the liver of control (simple fatty liver), immunoreactivity of 8-OHdG was weak in the nuclei of hepatocytes. Scale bar, 100 *μ*m in (**A**) and (**B**).

In the liver of patients with chronic hepatitis C, 8-OHdG immunoreactivity was strongly observed in the nuclei (weakly in the cytoplasm) of hepatocytes, Kupffer cells, and infiltrated lymphocytes (Figure 1A). The hepatocyte nuclei were differentiated from the nuclei of other cells using computed analyses at the point of nuclear shape and size. The number of 8-OHdG-positive hepatocytes in patients with chronic hepatitis C was counted from 7 to 123 cells per 10^5^ *μ*m^2^, the median being 42.5 cells per 10^5^ *μ*m^2^. Using the liver samples of patients with simple fatty liver as controls, immunoreactivity of 8-OHdG was faintly observed in the nuclei of hepatocytes in this experimental setting (Figure 1B). The specificity of the anti-8-OHdG antibody used in this study was confirmed by several parallel experiments. Sections in which the primary antibody was omitted or those treated with normal control serum instead of the primary 8-OHdG antibody consistently yielded negative staining. Localisation of 8-OHdG was considered specific because the recognition of hepatocytes was completely blocked by previous incubation with 25 ng ml^−1^ of 8-OHdG, but not by over a thousand-fold greater concentration of guanosine. Further, enzymatic treatment with RNase did not affect the immunoreaction of oxidised DNA.

### Analysis of factors associated with the occurrence of HCC in patients with chronic hepatitis C

**Figure 2:**
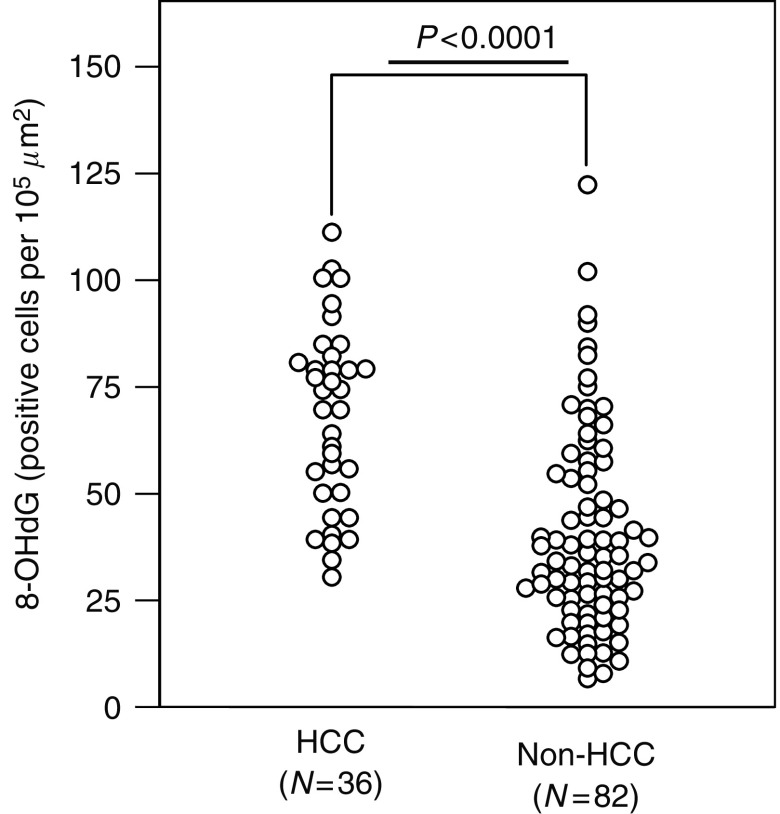
Comparison between 8-OHdG counts in patients who developed HCC (*N*=36) and those who remained free of HCC (non-HCC, *N*=82) during the follow-up period. Baseline 8-OHdG counts were significantly higher in the HCC group than in the non-HCC group in patients with chronic hepatitis C.

Until the end of follow-up (mean was 6.7±3.3 years), HCC occurrence was identified in 36 patients (30.5%) in this study. Seven patients died with no sign of HCC. The overall cumulative incidence of HCC was 3.4, 12.0, 17.2, and 38.9% at 1, 3, 5, and 10 years, respectively. To examine the effect of degree of liver oxidative DNA damage on HCC development during chronic HCV infection, clinical variables, including hepatic 8-OHdG quantification, were compared between patients who developed HCC and those who did not develop (non-HCC group) during the follow-up (Table 2). No significant difference was found in the patient age, body mass index, alcohol consumption, serum albumin levels, red blood cell count, and HCV genotype distribution between patients with and without HCC. In the group of patients with HCC, the proportion of male subjects, duration of infection, serum ALT, aspartate aminotransferase (AST), total bilirubin, hyaluronic acid, haemoglobin, iron, transferrin saturation, and ferritin levels at liver biopsy were significantly higher, and HCV-RNA titres and platelet count were significantly lower, than in the group of patients without HCC during the follow-up. The histological grading and staging scores were significantly higher in the HCC group than in the non-HCC group. The prevalence of hepatic iron deposits in patients with HCC was also significantly greater than that in non-HCC patients. Hepatic 8-OHdG expression levels in patients who developed HCC were significantly higher than in those who did not develop HCC (65.2±20.2 *vs* 40.0±23.5 positive cells per 10^5^ *μ*m^2^, *P*<0.0001; Mann–Whitney *U-*test) (Figure 2). When hepatic steatosis was evaluated by scoring system as 0, no steatosis; 1, <33% of hepatocytes with steatosis; 2, 33–66% of hepatocytes affected; 3, >66% of hepatocytes affected, the degree of steatosis was not significantly different between HCC and non-HCC groups.

**Figure 3:**
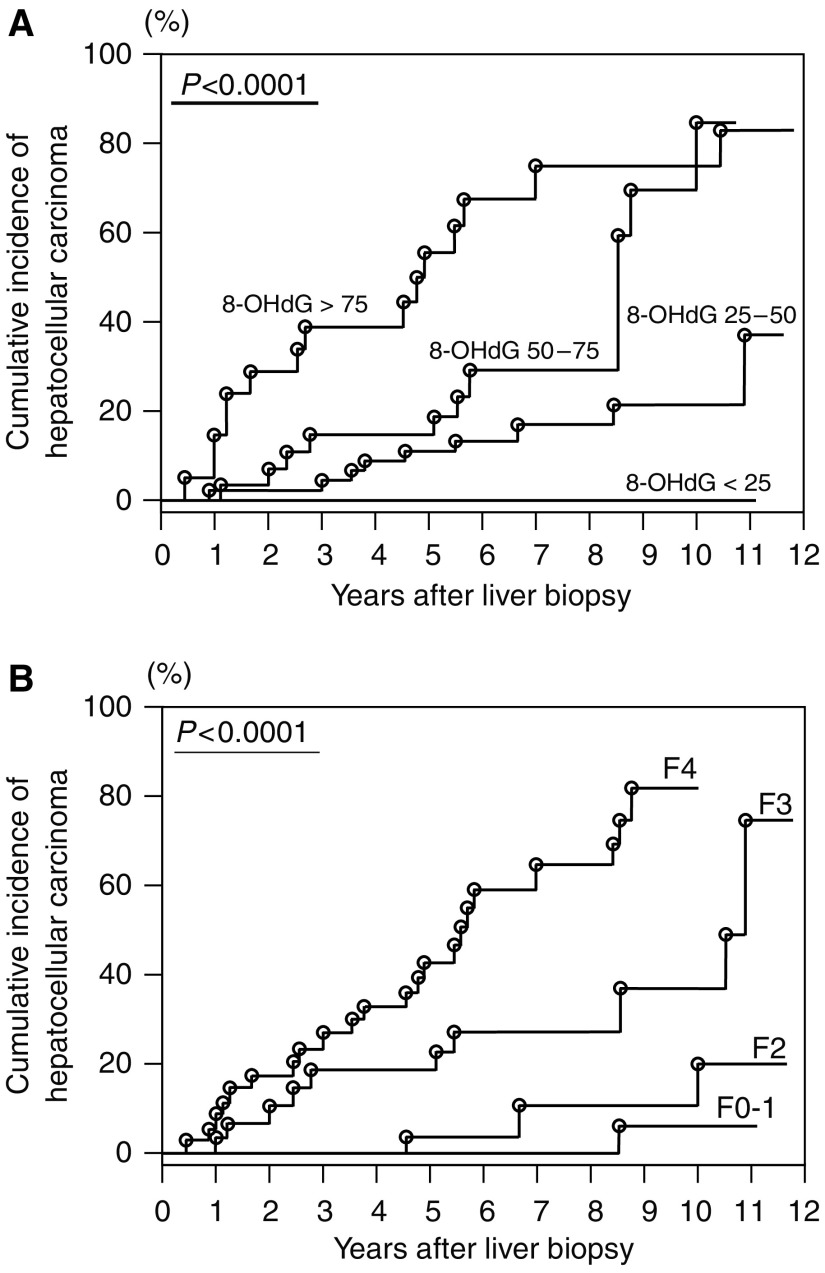
Cumulative incidence of HCC in 118 patients with chronic hepatitis C. Incidence curves were determined using the Kaplan–Meier method and statistical analysis was performed using the long-rank test. (**A**) Cumulative incidence of HCC divided by degrees of hepatic 8-OHdG expression levels. (**B**) Cumulative incidence of HCC divided by degrees of histological hepatic fibrosis staging score.

To examine the independent factors that affect the development of HCC, Cox proportional hazard regression analysis was performed using the 16 variables that were significantly different between HCC and non-HCC groups by univariate analyses. The multivariate analysis identified two factors as independent factors for HCC development: degree of hepatocytic 8-OHdG immunoreactivity (odds ratio, 1.487 (each 10 positive cells per 10^5^ *μ*m^2^ increase); *P*=0.0058) and histological staging (odds ratio, 4.090 (each stage 1 increase); *P*=0.0181) (Table 3). When the patients were stratified according to the degree of hepatic 8-OHdG counts and histological fibrosis staging, the cumulative incidence of HCC was significantly increased in proportion to these variables (long-rank test) (Figure 3A and B). The cumulative incidences of HCC of 3, 5, and 10 years were 0, 0, and 0% in 8-OHdG counts of <25 (cells per 10^5^ *μ*m^2^) subgroup (*n*=23), 4.4, 11.1, and 21.8% in 25–50 (cells per 10^5^ *μ*m^2^) subgroup (*n*=45), 14.2, 14.2, and 84.9% in 50–75 (cells per 10^5^ *μ*m^2^) subgroup (*n*=29), and 38.8, 55.5, and 74.6% in >75 (cells per 10^5^ *μ*m^2^) subgroup (*n*=21), respectively.

### Correlation between hepatocytic 8-OHdG counts and clinical characteristics in patients with chronic hepatitis C

**Figure 4:**
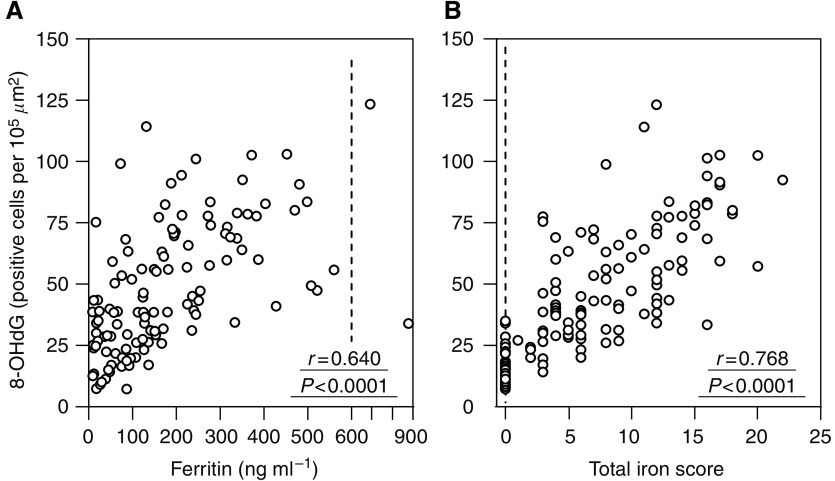
Correlations between hepatic 8-OHdG staining and serum ferritin levels (**A**), and TIS in hepatic tissues (**B**), in 118 patients with chronic hepatitis C.

To estimate the cause of hepatic oxidative DNA damage, correlation of clinical findings with hepatic 8-OHdG levels was evaluated (Table 4). The age of patients, body mass index, duration of infection, alcohol consumption, and the serum HCV-RNA titre were not related to the degree of oxidative DNA damage. 8-Hydroxydeoxyguanosine immunoreactivity was significantly higher in male than in female patients. Serum transaminases, platelet count, histological inflammation grade, and fibrosis stage were significantly correlated with the hepatic 8-OHdG levels. It is noteworthy that the hepatic 8-OHdG levels were strongly and positively correlated with body and hepatic iron deposition markers; serum ferritin levels and the hepatic iron deposit grade, that is, TIS, were strongly correlated with hepatic 8-OHdG count (8-OHdG *vs* ferritin, *r*=0.640, *P*<0.0001; *vs* TIS, *r*=0.768, *P*<0.0001) (Table 4 and Figure 4). These results suggest the association between hepatic 8-OHdG production and iron deposition in the liver of patients with chronic hepatitis C.

## DISCUSSION

Although free radicals are normally produced by many reactions essential for cell metabolism and energy production, they are also implicated in the pathogenesis of several different diseases (Valko *et al*, 2007). Reactive oxygen species production within the cells is controlled by numerous antioxidant intracellular defence mechanisms, but under certain conditions, ROS overproduction exceeds the cellular defences and damages cell components including nucleic acids (Valko *et al*, 2007). Reactive oxygen species attack on DNA causes the production of stable covalent bonds and the subsequent formation of DNA adducts, such as 8-OHdG (Shigenaga *et al*, 1989). Experiments in which DNA templates containing 8-OHdG were used indicated that this oxidatively modified DNA residue can induce G-C to T-A transversion at DNA replication (Kuchino *et al*, 1987; Shibutani *et al*, 1991), suggesting that the lesion is mutagenic and therefore potentially carcinogenic, but the role of this oxidative DNA adduct in human carcinogenesis is not entirely understood.

Chronic HCV infection is recognised as the most major risk factor for HCC (Nishioka *et al*, 1991; El-Serag, 2002; Seeff, 2002), but little is known about the precise role of HCC development in HCV-related liver disease. It was reported recently that oxidative damage is a peculiar feature of HCV-mediated liver injury. Patients with chronic hepatitis C showed increased oxidative stress markers in serum or in the liver (Shimoda *et al*, 1994; Sumida *et al*, 2000; Mahmood *et al*, 2004; Fujita *et al*, 2007). Therefore, we measured the amount of 8-OHdG in liver biopsy specimen of patients with chronic hepatitis C and examined its relation with future HCC development. Baseline clinical variables were compared between patients with and without HCC development. Based on univariate analysis, the following numerous variables were picked up for potential factors for HCC development: (1) gender and duration of infection, (2) hepatic inflammation (serum ALT and AST levels and histological grade), (3) hepatic fibrosis (hyaluronic acid, platelet count, and histological stage), (4) iron-related markers (haemoglobin, serum iron, transferrin saturation, ferritin, and TIS), (5) serum HCV-RNA titres, and (6) hepatocytic 8-OHdG counts. Cox proportional hazard analysis identified increased hepatic oxidative DNA damage, together with histological fibrosis, which is a well-recognised risk factor for HCC (Greten *et al*, 2005), as an independent risk factor for HCC development. This result suggests that the hepatic oxidative stress plays an important role in hepatocarcinogenesis and it may be a useful marker to predict future HCC development in chronic HCV-infected patients. Especially in the group of patients with hepatic 8-OHdG counts exceeding 75 positive cells per 10^5^ *μ*m^2^, the HCC incidence during the first 3 years was extremely high (38.8%) (Figure 3A), indicating that these patients constitute a very high-risk subgroup for developing HCC and should necessarily be carefully monitored by several modalities. Recently, Maki *et al* (2007) evaluated the expression levels of 8-OHdG of non-cancerous hepatic tissues in HCV-infected patients who developed HCC and received curative tumour resection. The postoperative cumulative HCC-free survival was significantly shorter in patients with the highest percentage of 8-OHdG-positive hepatocytes, indicating that the hepatic 8-OHdG levels are also useful for prediction of HCC recurrence in patients with chronic HCV infection who developed HCC.

Several additional risk factors for HCC were identified in patients with chronic hepatitis C in previous reports – increased age (Seeff, 2002), heavy alcohol intake (Donato *et al*, 1997), and chronic coinfection with HBV (Donato *et al*, 1997) – but our results did not identify these factors for HCC. This may be attributable to the fact that our study population excluded heavy alcohol abusers (defined as a chronic consumer of ethanol in excess of 40 g day^−1^ for male and 20 g day^−1^ for female for at least 5 years) and included relatively old patients (median age was 57.5 years). Patients coinfected with HBV were completely excluded from our study because all patients were seronegative for both hepatitis B surface antigen and hepatitis B core antibody. Recently, several reports have suggested that persons with diabetes mellitus are at an increased risk for developing HCC (El-Serag *et al*, 2001), and obesity, which frequently accompanies diabetes, has also been reported to increase the risk for hepatic steatosis and HCC in HCV-infected patients (Ohata *et al*, 2003). But, body mass index and hepatic steatosis were not significantly different between the HCC- and non-HCC-developed groups among our patients.

To determine the factors involved in the occurrence of hepatic oxidative stress during chronic HCV infection, epidemiologic, laboratory, and histological variables were examined for association with hepatocytic 8-OHdG staining counts. Quantitative analysis revealed that hepatocytic 8-OHdG levels were significantly correlated with serum aminotransferase levels and with the histological grading of necroinflammation, suggesting a possible link between hepatic oxidative stress and hepatic inflammation in chronic hepatitis C. It is unclear whether oxidative stress is the cause or the consequence of liver injury, but it has been demonstrated that oxidative stress can directly activate Kupffer cells, causing the release of inflammatory and profibrogenic cytokines such as tumour necrosis factor-*α* and transforming growth factor-*β* (Poli and Parola, 1997). Accordingly, the hepatocytic 8-OHdG counts were also significantly correlated with hepatic fibrosis, as assessed by the serum hyaluronic acid, platelet count, and histological staging score. Sumida *et al* (2000) have also shown a significant association between oxidative stress (serum thioredoxin levels) and hepatic fibrosis (hyaluronic acid, type IV collagen-7S domain, procollagen-III peptide) in HCV-positive persons, suggesting that ROS is an important cofactor in accelerating the development of hepatic fibrosis during chronic HCV infection, which may lead to further acceleration of HCC development. In addition, the hepatic 8-OHdG levels were significantly correlated with the serum ferritin and hepatic iron amounts assessed by TIS, suggesting a strong relationship between the damage to hepatocytic DNA and body store of iron in chronic hepatitis C patients. It is known that free iron promotes generation of oxygen radicals by catalysing the Fenton reaction in which Fe^2+^ reacts with H_2_O_2_ to generate highly reactive OH^•^ radicals, which can cause nucleic acid damage and 8-OHdG adducts. Therefore, iron may cause liver tissue injury by increasing the formation of toxic hydroxyl radicals leading to progression of liver inflammation, fibrosis, and increased risk for developing liver cancer during chronic HCV infection. Increased iron stores were associated with increased oxidative DNA damage, suggesting that removing iron stores in the body, for example by phlebotomy (Yano *et al*, 2004), or dietary iron restriction (Iwasa *et al*, 2004), which has been accepted as a useful treatment option, may delay or reduce the incidence of HCC in patients with chronic hepatitis C. Additional studies are warranted to determine whether these iron-reduction therapies are effective for reducing HCC and for improving the clinical outcomes of patients with chronic HCV infection.

In conclusion, this study clearly showed that in patients with chronic hepatitis C, the oxidative DNA damage in the liver frequently occurred and that it was strongly associated with increased risk for HCC. Strong positive correlations between hepatic oxidative stress and iron overload suggest that iron content may be a mediator of hepatic oxidative stress and that iron reduction may be beneficial to reduce the HCC incidence in chronic HCV-infected patients.

## Figures and Tables

**Table 1:** Baseline characteristics of patients with chronic hepatitis C

**Characteristics**	**Chronic hepatitis C (*N*=118)**
Age (years)	55.8±10.8 (57.5)
Gender (M/F)	66/52
	
*Laboratory data*	
ALT (IU l^−1^)	73.5±53.4 (58.0)
AST (IU l^−1^)	70.1±41.8 (61.5)
Platelet count ( × 10^4^ mm^−3^)	14.9±5.9 (14.6)
Serum HCV-RNA (kIU ml^−1^) (*N*=89)	1570±1240 (1420)
HCV genotype (1a/1b/2a/2b) (*N*=60)	0/53/5/2
	
*Liver histology*	
Inflammatory activity (0/1/2/3)^a^	1/41/49/27
Fibrosis staging (0/1/2/3/4)^b^	1/29/26/27/35
Total iron score^c^	7.75±5.80 (7.00)
8-OHdG-positive hepatocytes (per 10^5^ *μ*m^2^)	48.4±26.2 (42.5)

Data are expressed as mean±s.d. (median).

ALT=alanine aminotransferase; AST=aspartate aminotransferase; HCV=hepatitis C virus; 8-OHdG=8-hydroxydeoxyguanosine.

a Inflammatory activity was graded according to the intensity of necroinflammatory lesions: 0, no histological activity; 1, mild activity; 2, moderate activity; 3, severe activity.

b Fibrosis staging was scored as follows: 0, no fibrosis; 1, portal fibrosis without septa; 2, portal fibrosis with few septa; 3, numerous septa without cirrhosis; 4, cirrhosis.

c The histological quantification of iron was assessed by total iron score proposed by Deugnier *et al* (1992).

**Table 2:** Comparison of epidemiologic and clinical variables of patients who developed HCC and patients who remained free of HCC during the follow-up period

**Characteristics**	**HCC group (*N*=36)**	**Non-HCC group (*N*=82)**	***P*-value**
Age (years)	57.3±8.2	54.7±11.4	0.3718^a^
Gender (M/F)	26/10	40/42	**0.0182** b
Body mass index (kg m^−2^)	23.6±3.5	24.1±3.2	0.6657^a^
Duration of HCV infection (years) (*N*=58)	31.7±10.5	26.9±9.8	**0.0463** a
Alcohol intake (g day^−1^)	21.0±37.0	21.2±38.9	0.6221^a^
			
*Laboratory data*			
ALT (IU l^−1^)	91.9±50.4	65.6±52.9	**0.0021** a
AST (IU l^−1^)	91.4±42.7	60.5±38.3	**0.0003** a
Serum albumin (g dl^−1^)	3.65±0.40	3.75±0.45	0.1235^a^
Total bilirubin (mg dl^−1^)	0.96±0.29	0.75±0.88	**<0.0001** a
Hyaluronic acid (ng ml^−1^)	206±138	132±151	**0.0003** a
Platelet count ( × 10^4^ mm^−3^)	11.7±4.5	16.4±5.8	**<0.0001** a
Red blood cell count ( × 10^4^ mm^−3^)	429±48	418±50	0.1993^a^
Haemoglobin (g dl^−1^)	13.9±1.3	13.2±1.6	**0.0302** a
Serum iron (*μ*g dl^−1^)	151±68	121±62	**0.0320** a
Transferrin saturation (%)	45.7±22.6	36.2±20.0	**0.0289** a
Serum ferritin (ng ml^−1^)	264±158	151±149	**0.0002** a
Serum HCV-RNA (kIU ml^−1^) (*N*=89)	844±900	1720±1260	**0.0068** a
HCV genotype (1a/1b/2a/2b) (*N*=60)	0/5/2/0	0/48/3/2	0.1100^b^
			
*Liver histology*			
Inflammatory activity (0/1/2/3)^c^	0/4/18/14	1/37/31/13	**0.0015** b
Fibrosis staging (0/1/2/3/4)^d^	0/1/3/10/22	1/28/23/17/13	**<0.0001** b
Total iron score^e^	11.09±4.75	6.23±5.62	**<0.0001** a
8-OHdG-positive hepatocytes (per 10^5^ *μ*m^2^)	65.2±20.2	40.0±23.5	**<0.0001** a

Data are expressed as mean±s.d.

HCC=hepatocellular carcinoma; ALT=alanine aminotransferase; AST=aspartate aminotransferase; HCV=hepatitis C virus; 8-OHdG=8-hydroxydeoxyguanosine.

a Mann–Whitney *U*-test.

b Fisher's exact test, otherwise *χ*^2^ test.

c Inflammatory activity was graded according to the intensity of necroinflammatory lesions: 0, no histological activity; 1, mild activity; 2, moderate activity; 3, severe activity.

d Fibrosis staging was scored as follows: 0, no fibrosis; 1, portal fibrosis without septa; 2, portal fibrosis with few septa; 3, numerous septa without cirrhosis; 4, cirrhosis.

e The histological quantification of iron was assessed by total iron score proposed by Deugnier *et al* (1992).

**Table 3:** Factors associated with the occurrence of HCC in patients with chronic hepatitis C by Cox proportional hazard regression analysis

**Factor**	**Odds ratio**	**95% CI**	***P*-value**
Count of 8-OHdG-positive hepatocytes (each 10 cells per 10^5^ *μ*m^2^ increase)	1.487	1.12–1.97	0.0058
Fibrosis staging (each stage 1 increase)	4.090	1.27–13.15	0.0181

HCC=hepatocellular carcinoma; 8-OHdG=8-hydroxydeoxyguanosine; CI=confidence interval.

**Table 4:** Correlations between clinical findings and 8-OHdG levels in the liver of patients with chronic hepatitis C (*N*=118)

		**Statistics**	
**Characteristics**	**Hepatic 8-OHdG levels (positive cells per 10^5^ *μ*m^2^)**	***r***	***P***-**values**
Age (years)		0.149^a^	0.1059^a^
Gender			
Male (*N*=66)	57.7±23.3		**<0.0001** b
Female (*N*=52)	36.7±22.4		
Body mass index (kg m^−2^)		0.073^a^	0.4271^a^
Duration of HCV infection (years) (*N*=58)		0.237^a^	0.0677^a^
Alcohol intake (g day^−1^)		0.121^a^	0.2709^a^
			
*Laboratory data*			
ALT (IU l^−1^)		0.541^a^	**<0.0001** a
AST (IU l^−1^)		0.605^a^	**<0.0001** a
Platelet count ( × 10^4^ mm^−3^)		−0.430^a^	**<0.0001** a
Serum ferritin (ng ml^−1^)		**0.640** a	**<0.0001** a
Serum HCV-RNA (kIU ml^−1^) (*N*=89)		−0.197^a^	0.0721^a^
			
* Inflammatory activity* c			
A0 or A1 (*N*=42)	32.2±21.2		
A2 (*N*=49)	52.3±22.6		**<0.0001** d
A3 (*N*=27)	62.4±26.0		
			
* Fibrosis staging* e			
F0 or F1 (*N*=30)	26.6±14.7		
F2 (*N*=26)	46.0±22.1		**<0.0001** d
F3 (*N*=27)	52.4±21.2		
F4 (*N*=35)	66.3±26.3		
Total iron score^f^		**0.768** a	**<0.0001** a

Data are expressed as mean±s.d.

8-OHdG=8-hydroxydeoxyguanosine; HCV=hepatitis C virus; ALT=alanine aminotransferase; AST=aspartate aminotransferase.

a Spearman rank correlation test.

b Mann–Whitney *U*-test.

c Inflammatory activity was graded according to the intensity of necroinflammatory lesions: 0, no histological activity; 1, mild activity; 2, moderate activity; 3, severe activity.

d Kruskal–Wallis test.

e Fibrosis staging was scored as follows: 0, no fibrosis; 1, portal fibrosis without septa; 2, portal fibrosis with few septa; 3, numerous septa without cirrhosis; 4, cirrhosis.

f The histological quantification of iron was assessed by total iron score proposed by Deugnier *et al* (1992).
